# Weak latitudinal gradients in insect herbivory for dominant rangeland grasses of North America

**DOI:** 10.1002/ece3.6374

**Published:** 2020-05-26

**Authors:** Dylan R. Kent, Joshua S. Lynn, Steven C. Pennings, Lara A. Souza, Melinda D. Smith, Jennifer A. Rudgers

**Affiliations:** ^1^ Department of Biology University of New Mexico Albuquerque NM USA; ^2^ Department of Biology and Biochemistry University of Houston Houston TX USA; ^3^ Oklahoma Biological Survey & Department of Microbiology and Plant Biology University of Oklahoma Norman OK USA; ^4^ Department of Biology Colorado State University Fort Collins CO USA; ^5^ Present address: Department of Biology University of Bergen Bergen Norway

**Keywords:** biogeography, climate change, grass, herbivory, latitudinal gradient, plant–insect interactions, rangeland

## Abstract

Patterns of insect herbivory may follow predictable geographical gradients, with greater herbivory at low latitudes. However, biogeographic studies of insect herbivory often do not account for multiple abiotic factors (e.g., precipitation and soil nutrients) that could underlie gradients. We tested for latitudinal clines in insect herbivory as well as climatic, edaphic, and trait‐based drivers of herbivory. We quantified herbivory on five dominant grass species over 23 sites across the Great Plains, USA. We examined the importance of climate, edaphic factors, and traits as correlates of herbivory. Herbivory increased at low latitudes when all grass species were analyzed together and for two grass species individually, while two other grasses trended in this direction. Higher precipitation was related to more herbivory for two species but less herbivory for a different species, while higher specific root length was related to more herbivory for one species and less herbivory for a different species. Taken together, results highlight that climate and trait‐based correlates of herbivory can be highly contextual and species‐specific. Patterns of insect herbivory on dominant grasses support the hypothesis that herbivory increases toward lower latitudes, though weakly, and indicates that climate change may have species‐specific effects on plant–herbivore interactions.

## Graphical abstract

We investigated latitudinal patterns of insect herbivory in dominant North American rangeland grasses, finding support for the hypothesis that herbivory increases toward lower latitudes. Precipitation and plant functional traits were predictive of herbivory but in a species‐specific manner. Together, results suggest that geographic variation in herbivory is related to climate, soil, and plant traits in a species‐specific manner.

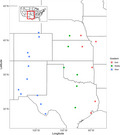

## 1 INTRODUCTION

Ecologists have long predicted that plant–herbivore interactions are strongest at low latitudes (Coley & Barone, 1996; Schemske, Mittelbach, Cornell, Sobel, & Roy, 2009). This hypothesis arose from Dobzhansky's idea (1950) that a relatively stable and favorable abiotic environment amplifies the relative importance of biotic interactions near the equator as compared to high latitudes, where a harsh abiotic environment and strong seasonality most strongly limit population dynamics (see also MacArthur, 1972). The assertion that biotic interactions are more intense at low than high latitudes underlies many hypotheses for latitudinal gradients in species diversity (Dobzhansky, 1950; Moles, Bonser, Bonser, Poore, Wallis, & Foley, 2011). However, while some studies of herbivory have documented the predicted latitudinal gradient in herbivory, several others have not (reviewed by Moles, Bonser, et al., 2011).

Focusing on insect herbivory, the current literature on latitudinal gradients in insect herbivory presents contradictory patterns. Some studies observe the predicted increase in insect herbivory toward low latitudes (e.g., Pennings & Silliman, 2005), while others support the opposite pattern (e.g., Adams & Zhang, 2009), a nonlinear pattern (e.g., Kim, 2014), or no latitudinal pattern (e.g., Andrew & Hughes, 2005; Lynn & Fridley, 2019). In addition, some studies report that the existence of a gradient depends on the herbivore or plant species identity (Anstett, Naujokaitis‐Lewis, & Johnson, 2014; Lim, Fine, & Mittelbach, 2015). Some evidence suggests that latitudinal patterns may differ even among co‐occurring plant species (Kim, 2014). These findings highlight the lack of consensus on whether an equatorial peak in insect herbivory intensity is a universal trend. In addition, research has been limited to relatively few ecosystems and plant clades, constraining the ability to generalize a global pattern.

Several factors may explain variation among the results of past studies. Nonuniversal sampling methods and a general lack of consistency in methodology may play a large role in generating divergent results (Andrew, Roberts, & Hill, 2012; Moles, Bonser, et al., 2011; Pennings & Silliman, 2005). One key methodological issue is that many studies fail to account for latitudinal differences in phenology during sampling (e.g., Pennings & Silliman, 2005). If insect herbivory at poleward sites is recorded at an earlier phenological stage than at equatorial sites, results could be biased toward the detection of the expected gradient. Alternatively, the longer growing season at low latitudes makes it important to consider the rate of insect herbivory or cumulative damage (Andrew & Hughes, 2005). Some studies examine different plant taxa at different latitudes, preventing the separation of latitudinal pattern from plant species identity (see Pennings et al., 2007). Furthermore, most prior studies sampled a single latitudinal gradient (but see Gao, Fang, & Zhao, 2019). The lack of spatial replication can make it difficult to disentangle the effects of latitude from other correlated factors, such as nutrient availability or land use change.

Environmental factors (e.g., precipitation and soil nutrients) and plant traits (e.g., leaf toughness and nutrient content) typically do not follow a simple latitudinal gradient, and these factors may have important roles in driving biogeographic patterns in plant–herbivore interactions (Andrew & Hughes, 2005; Kim, 2014; Moreira, Abdala‐Roberts, Parra‐Tabla, & Mooney, 2015). For example, 66% of the latitudinal variation in insect herbivore damage was explained by precipitation and mean annual temperature in one study (Garibaldi, Kitzberger, & Ruggiero, 2011). Although latitude can be a useful integrator of several axes of global variation in climate, relatively few studies investigate the hypothesized underlying climatic drivers behind latitudinal patterns in herbivory (e.g., Adams & Zhang, 2009; Gao et al., 2019; Moreira et al., 2015; Zhang, Zhang, & Ma, 2016), while even fewer consider nutrient availability that can have different spatial patterns than climate variables (e.g., Lynn & Fridley, 2019; Moreira, Castagneryrol, Abdala‐Roberts, & Berny‐Mier y Teran JC, Timmermans BGH, Bruun HH, Covelo, F, Glauser G, Rasmann S, Tack AJM., 2018). In addition, it is possible that latitudinal variation in insect herbivore damage and plant resistance to herbivory are driven by resource availability, trade‐offs in plant growth and defenses (Kim, 2014), and that they depend on herbivore specialization (Dyer & Forister, 2019), as well as land use or urbanization (Just, Dale, Long, & Frank, 2019). Though climate is surely a strong driver of latitudinal variation in herbivory, deviations from this expected pattern are likely due to unmeasured edaphic and plant‐trait controls on consumption rates.

Grasslands provide interesting systems for evaluating latitudinal gradients in plant–insect herbivore interactions, given grasses are historically hypothesized to have evolved grazing tolerance strategies (e.g., overcompensation; McNaughton, 1983) and are typically less chemically defended than other plant clades (Gibson, 2009). While silica may serve as an herbivore‐induced deterrent against tissue loss (Hartley & DeGabriel, 2016), grasses may often recover from large biomass loss to grazing by enhanced regenerative growth (McNaughton, 1983) rather than avoid or deter smaller amounts of insect herbivory with high chemical defense investment. In temperate grasslands lacking large grazers, insects are the dominant herbivores. Grasshoppers alone, which vary greatly in their feeding patterns and preferences (Joern, ), can be responsible for a large proportion of foliar damage (Tscharntke & Greiler, 1995) and affect grassland ecosystem functioning (Belovsky & Slade, 2017; LaPierre, Joern, & Smith, 2015). Grasses have received relatively little study with respect to latitudinal gradients in insect herbivory, despite their global importance (Shantz, 1954). To our knowledge, no previous study has tested whether latitudinal gradients occur for the dominant plant species in terrestrial grassland ecosystems.

**Figure 1:**
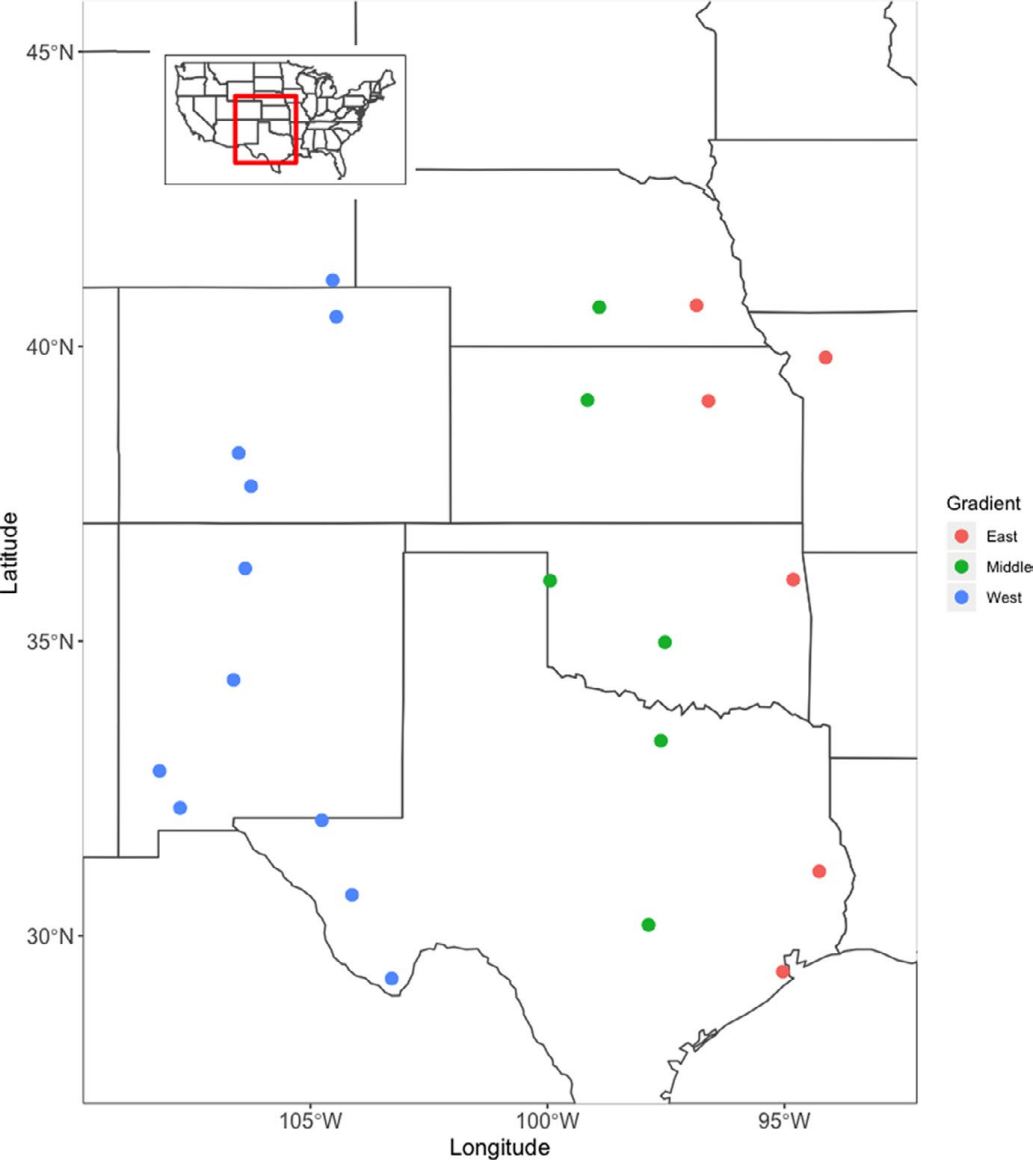
Map of sampling locations across the North American plains indicating the gradient of sampling and an inset of the study region within the greater continental USA. Geographic coordinates and further site details are provided in Table S1

We evaluated latitudinal gradients in insect herbivory across grasslands of the Great Plains of North America. Hereafter, “herbivory” refers to insect herbivory and we focused on leaf area lost to herbivory. Therefore, damage by cell sucking, sap‐feeding, and mining insects was unaccounted for. We assessed damage on five dominant grass species over a major climate transition (10 degrees of latitude, 14 degrees of longitude), and we replicated latitude by surveying three independent latitudinal gradients (Figure 1). We also evaluated the relative importance of selected climate variables, edaphic factors, and plant traits as drivers of geographic variation in herbivory.

## 2 MATERIALS AND METHODS

### 2.1 Study sites

We sampled 23 sites on three independent latitudinal gradients; each of which spanned ~10° of latitude and represented a different major ecoregion of the Great Plains. The *West* gradient was characterized by shortgrass prairie and desert grassland, the *Middle* gradient was largely mixed‐grass prairie, and the *East* gradient was tallgrass prairie. Our latitudinal range was comparable to that of similar studies (e.g., Andrew & Hughes 2005; Adams & Zhang, 2009; Kim, 2014) and encompassed substantial climate variation, with mean annual temperature ranging ~10°C north–south and precipitation varying ~1,000 mm east–west (Shafer et al. 2014). Specifically, our study spanned a gradient of 197 mm to 1,001 mm in 30‐year normal mean annual precipitation (MAP) and 2341C to 5837C in mean growing degree days (GDD). We sampled a total of 23 sites: 11 in the *West*, and six each in the *Middle* and *East* (see Figure 1). At the sites CPR, HAR, HPG, KNZ, and SEV, we sampled a second location within the same landscape from controls plots of an ongoing experiment (EDGE; http://edge.biology.colostate.edu/index.html; Table S1). Most sites occurred in national or local preserves that had not been grazed by large vertebrate herbivores. However, sites CAD and DMT were likely grazed by cattle, although we sampled from locations that had no evidence of recent grazing so that no herbivory estimates included cattle damage. Some damage observed in our survey may have been caused by small rodents or other small vertebrates.

### 2.2 Focal plant species

We sampled five perennial C4 grasses: blue grama (*Boutelou gracilis*) and buffalo grass (*B. dactyloides*; formerly genus *Buchloë*), both ubiquitous in shortgrass prairie; black grama (*B. eriopoda*), which dominates desert grasslands; big bluestem (*Andropogon gerardii*), an abundant species in tallgrass prairie; and little bluestem (*Schizachyrium scoparium*), which is common in both tallgrass and mixed‐grass prairies. *B. gracilis* and *S. scoparium* were the most widely sampled.

### 2.3 Latitudinal survey

During summer 2015, at each site, we sampled twelve individual plants per species. We closely examined two haphazardly chosen live, fully expanded leaves per individual. Because all species were not present at every site, the number of sites sampled varied among plant species (see Table S1). Individuals were selected as the nearest plant every 10 m along five transects spaced at 10‐m intervals (within a sampling area of ~50 m × 50 m). For most sites, we used a separate sampling grid for each grass species due to nonoverlapping species distributions at the local scale. Following standard methods for herbivory assessment (Pennings et al., 2007), we visually estimated the percentage of leaf area missing from each of two randomly selected leaves per plant and we focused our sampling attention on chewing insect damage. Instead of binning damage estimates into categories (e.g., 11%–25%), as in Pennings et al. (2007), values were recorded as continuous variation from 0% to 50% (generally scored to the nearest 5%) or scored as 75% damage for all leaves damaged by >50%. While this latter category may have slightly inflated our estimates, only 18 leaves of 1,618 were scored as >50% damaged. Maximum damage observed was ~100%. A consequence of sampling on a large geographic scale at similar phenology was that multiple observers were required for data collection. Prior to sampling, all observers calibrated their estimates of herbivore damage in the field to maintain consistency. For analysis, we averaged herbivore damage between the two leaves per individual plant.

To help control for phenological differences among plants at different latitudes, we sampled all sites at similar growing degree days (GDD) based on the 30‐year climate average (2,680 ± 418 s.d. degree days, using a 0°C base). This ensured that leaves from different sites were sampled at the same relative age. Sample dates appear in Table S1.

Per field observations, grasshoppers were a dominant component of the insect herbivore community in our system. Grasshoppers can experience periodic outbreaks and vary greatly in population size over time (Tscharntke & Greiler, 1995); thus, it is possible that results obtained during another year might differ from our study. We focused sampling effort on coverage of a large geographic area at the expense of collecting data over multiple timepoints. However, we had no indications that herbivore abundance was anomalous in 2015. For the two sites for which we had grasshopper count data, abundance during 2015 was within 12%–13% of the long‐term mean. At the Sevilleta Long Term Ecological Research (LTER) black grama site in 2015, average grasshoppers per ha was 309 ± 23.2 *SE*, and the long‐term (1992–2015) average was 276 ± 7.5 *SE*. At the Sevilleta LTER blue grama site, mean grasshopper density per ha in 2015 was 357 ± 8.9 *SE* and the long‐term (2002–2015) mean was 411  ±28.7 *SE*.

### 2.4 Abiotic factors

We examined six abiotic factors as possible correlates of herbivory. Two were climatic: growing season precipitation and cumulative GDD. The other four were edaphic: soil nitrogen (as nitrate), phosphorous, pH, and organic matter (SOM). For precipitation and GDD, we defined the growing season as March through October. We used a baseline temperature of 0°C for GDD, as is typical for perennial grasses (Henebry, 2013). We created climate windows for each factor over three separate time series, allowing us to determine whether variation in herbivory was best explained by current, short‐, or long‐term climate data. We used the year of field sampling (2015), the average of the three most recent years (2013–2015), or the 30‐year average (including 2015). We extracted climate data at the 800 m spatial resolution using the PRISM database (PRISM Climate Group 2015). The other four abiotic factors were related to edaphic conditions: nitrogen (as nitrate), phosphorous, pH, and soil organic matter (SOM). We collected soil samples in situ, taking 10–20 g from beneath each plant. Samples were combined to obtain a single value per edaphic factor for each species × site combination. Soil phosphorous and pH were determined using protocols in Robertson, Coleman, Bledsoe and Sollins (1999). SOM was determined using the loss on ignition method by Zhang and Wang (2014). Soil ammonium and nitrate were determined calorimetrically using the Lachat Autoanalyzer QuikChem method 12‐107‐06‐1‐A and 12‐107‐04‐1‐*F* (Loveland, CO).

### 2.5 Plant traits

We assessed specific leaf area (SLA) and specific root length (SRL) as possible correlates of herbivory. SLA and SRL are above‐ and belowground indicators of resource acquisition trade‐offs (Pérez‐Harguindeguy et al., 2013) but are not typically examined in studies of latitudinal variation in foliar herbivory; high SLA and SRL indicate high resource acquisition investment and low tissue longevity (Reich, Walters, & Ellsworth, 1992). Traits were measured on the same individuals sampled in the latitudinal survey following published protocols (Pérez‐Harguindeguy et al., 2013). Whole live plants were pressed in the field immediately after herbivore damage assessments, and two leaves were used for measurement per individual. For SLA, we rehydrated the dried leaves that had been stored in a plant press by placing individual leaf samples in separate, sealed petri dishes with ~100 ml of water. Samples were stored at room temperature during rehydration period (~48 hr). Rehydrated leaves were scanned and digitized for total area (cm^2^) using WinFOLIA (Regent Instruments Inc., Canada). After measuring leaf area, leaves were oven‐dried at 65°C for ~48 hr and then weighed for mass. SLA was calculated as rehydrated leaf area divided by leaf mass (cm^2^/g). Literature suggests that measuring SLA for live plants is preferred (Tomaszewski & Górzkowska, 2016), but this was not feasible in our study due to the sampling schedule required to collect data at numerous sites over a large geographic area and control for phenology. For SRL, a subsample of fine roots (ca. 10) from each individual (12 in total) from the field collections were dug up then stored in 50% ethanol. For imaging, roots were submerged in a small amount of DI water in a clear plastic tray with individual roots teased apart. Units of total root length were determined using WinRHIZO (Regent Instruments Inc.). After imaging, roots were oven‐dried at 65°C for ~48 hr and then weighed for specific mass. SRL was calculated as total root length divided by mass (cm/g).

### 2.6 Statistical analysis

#### 2.6.1 Latitudinal gradients

We used a linear mixed effects model with herbivory as a function of latitude. We averaged herbivory data per species per site prior to analyses because high zero inflation of the individual‐level data made the choice of error distribution difficult. Additionally, climate and edaphic variables were measured at the population level, rather than the individual level. We first constructed an all species model where herbivory for all species was analyzed together with species, site, and gradient random effects. For every all species model (for latitude and other dependent variables), we checked whether using random slopes for species improved model fit, and it did not (Table S6). We then analyzed latitudinal patterns for each species separately with the random effect of replicate gradient (West, East, Central), except for *B. eriopoda* which only occurred in one gradient. Random intercept variance terms for each model are reported in Table S5). Models were run with *lmer* in the “lme4” package (Bates, Maechler, Bolker, & Walker, 2015; R Core Team 2019). To improve homogeneity of variances, we logit‐transformed herbivore damage estimates prior to analysis, which when back‐transformed appear as nonlinear relationships in units of measurement in our graphics. We evaluated parameter fit with Wald chi‐square tests. *Bouteloua eriopoda* was analyzed using the “lm” function in base R (R Core Team 2019), given the lack of the random effect of replicated gradient, and model fit was evaluated with an *F* test.

#### 2.6.2 Abiotic factors and plant traits

First, we evaluated the predictive ability of different climate windows for GDD and precipitation from the year of sampling (2015), average of years 2013–2015, and 30‐year average by constructing linear mixed effects models similar to the above models. The respective climate variables were substituted for latitude as predictors. We compared model fit between climate windows using *AICc* and weights with the *MuMIn* package (Barton, 2015). Models with lower *AICc* values have greater within sample predictive ability (Burnham & Anderson 2002; Anderson, 2008).

To determine the relative importance of abiotic factors and traits in explaining geographic patterns in herbivory, we used similar models as described above, except with an abiotic predictor or plant trait rather than latitude. We used the three‐year GDD and 2015 precipitation climate windows because prior analyses suggested these were better predictors of variation in herbivory than the alternative time windows we tested: year of sampling (2015), average of years 2013–2015, and 30‐year average (Table S3). Prior to analysis, we standardized all predictors to a mean of zero and a standard deviation of one to allow for the comparison of slope estimates as effect sizes. At 4 of 23 sites (RNF, GNF, FMT, and CNF), species were missing data on specific root length due to limited root tissue. We interpolated the 4 missing observations using species‐specific regression of SRL on latitude. We developed models for each climatic, edaphic, and trait variable separately to avoid over‐parameterization and variance inflation due to multicollinearity among predictors. We carried out model selection using *AICc* to compare models of climate, edaphic, or trait correlates of herbivory as above; this process ranks the relative importance of alternative correlates of herbivory.

## 3 RESULTS

### 3.1 Latitudinal gradients

**Figure 2:**
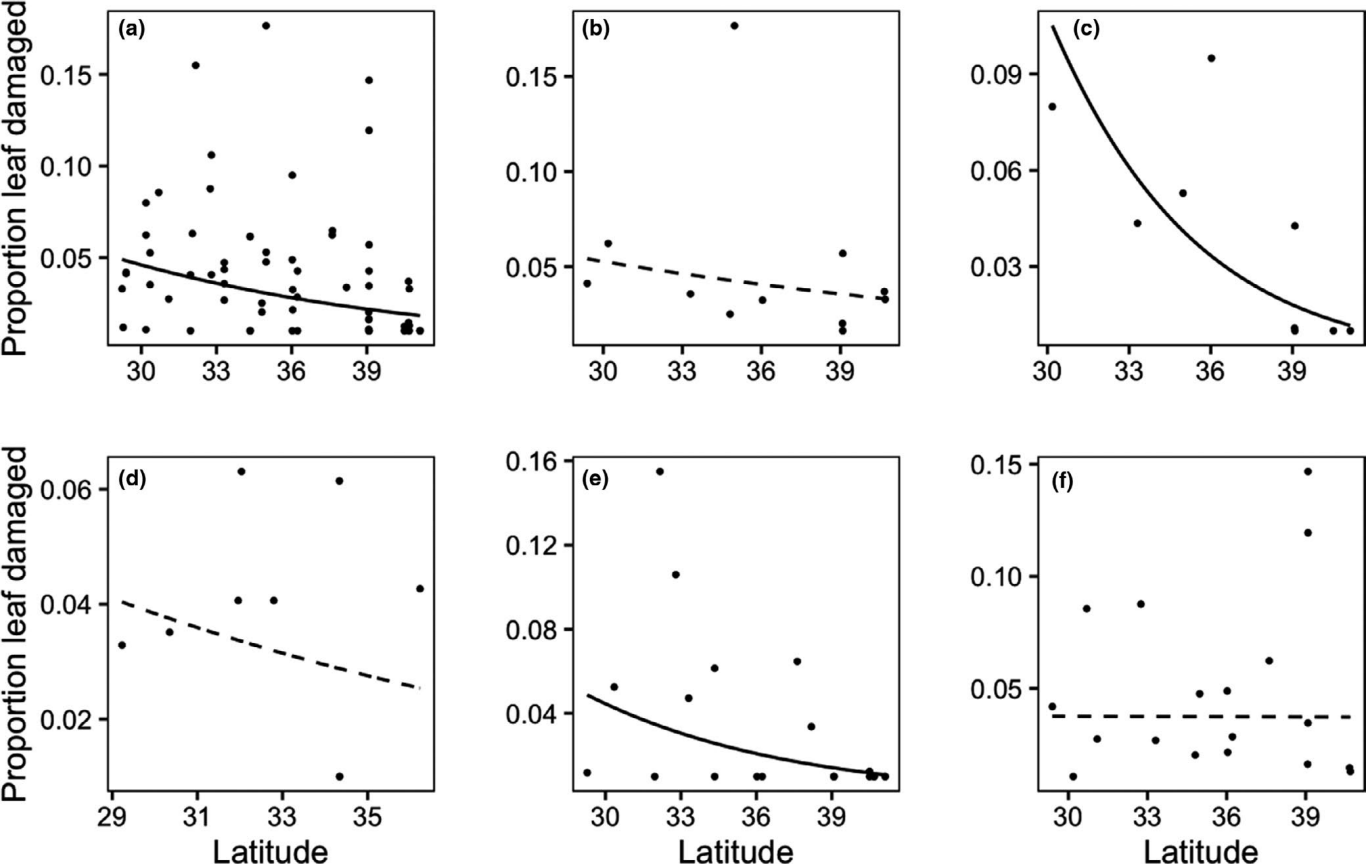
Percentage herbivore damage (mean per site) over latitude for all species combined and for each focal species individually. Each point represents average damage for at species × site combination. A solid best‐fit line indicates a relationship with latitude for which *p* < .05 and a dashed line represents a nonsignificant trend (0.98 > *p*> .05). (a) All species, (b) *Andropogon gerardii* (c) *Bouteloua dactyloides*, (d) *Bouteloua eriopoda*, (e) *Bouteloua gracilis*, and (f) *Schizachyrium scoparium*

Herbivory increased toward lower latitudes, although the significance of this relationship was dependent on species (Figure 2). Damage increased at low latitudes for all species analyzed together, as well as for *B. dactyloides* and *B. gracilis* individually. Though the other species lacked significant relationships, *A*. *gerardii* and *B*. *eriopoda* trended toward more herbivory at lower latitudes. Complete statistical results are in Table S2.

### 3.2 Abiotic factors and plant traits

There was little evidence that using a different time window of climate data improved predictions of herbivory, given there was little (<2) difference in *AICc* values among models (Table S3). Timescale comparisons determined that short‐term (2015) precipitation best explained variation in herbivory. The models including precipitation in 2015 or averaged over the prior three years better predicted herbivory than the 30‐year average for all five species, though not in the all species model (Table S3). In contrast, the explanatory power of GDD was not clearly different among the year of data collection (2015), the short‐term average (3‐year), or 30‐year GDD average. The 30‐year average was best for two species, 3‐year average was best for two species, and 2015 GDD was best for one species. Given these results, the following model comparisons use only 2015 precipitation and three‐year GDD.

**Figure 3:**
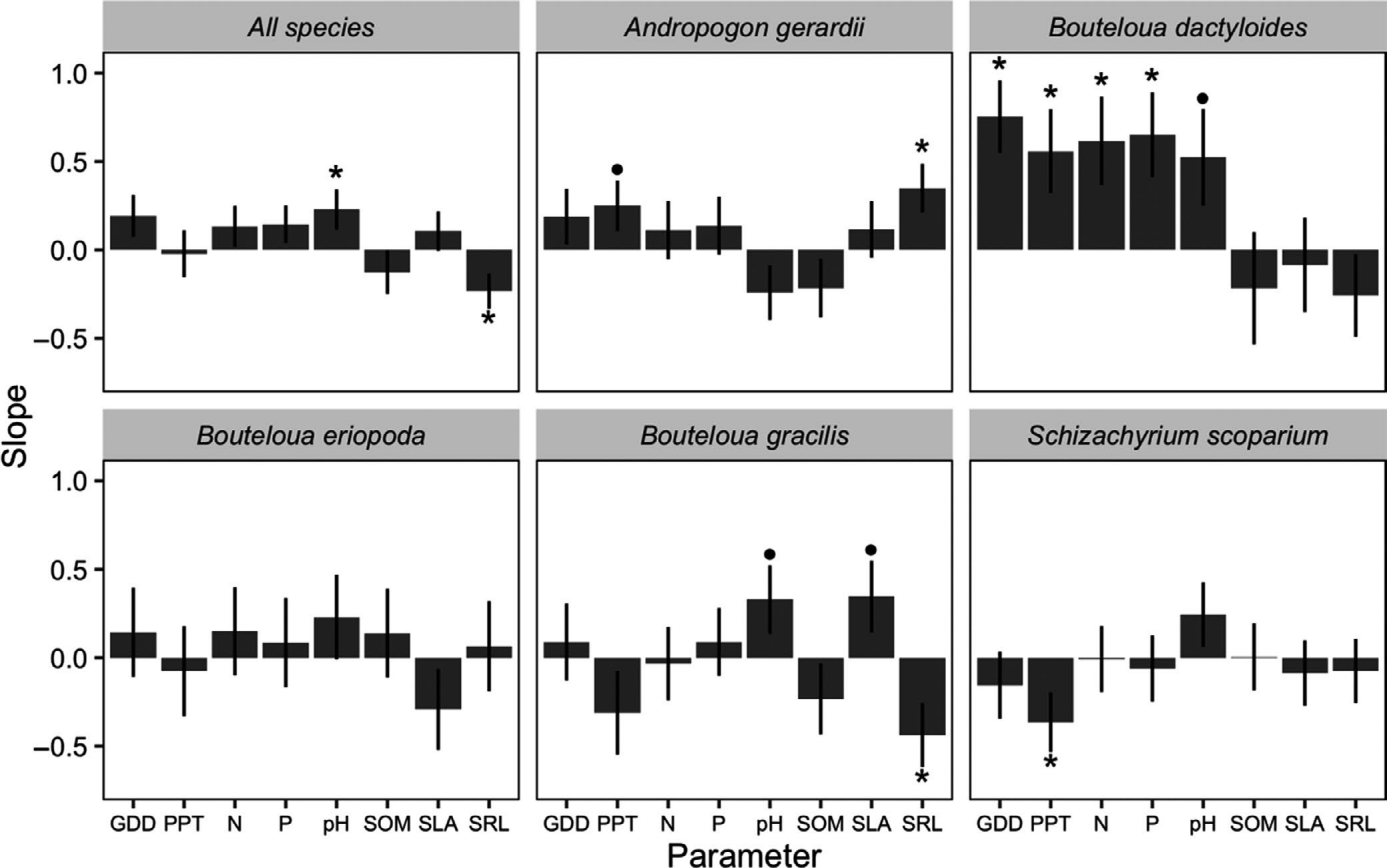
Relative effects (standardized slopes) of abiotic factors (growing degree days as *GDD*, growing season precipitation (*PPT*), nitrogen (*N*), phosphorous (*P*), pH, soil organic matter (*SOM*), specific leaf area (SLA), or specific root length (SRL) on herbivore damage for all species combined, and for each focal species individually. A positive value indicates an increase in herbivory with the factor, *indicates *p* < .05., and • indicates 0.1 > *p*> .05

Herbivory tended to increase with hotter temperatures, as captured by more GDD (four of five species), as well as with more precipitation (four out of five species), and higher levels of soil pH (four of five species; Figure 3). Across all species, the best predictor of damage was soil pH and SRL, with more herbivory in basic than acidic soils and lower SRL. Herbivore damage was well predicted by precipitation for some species, but the direction varied. Herbivory increased at sites with greater precipitation in *A*. *gerardii* and *B*. *dactyloides* but declined at sites with greater precipitation in *S. scoparium* (Figure 3). Similarly, SRL was often a significant predictor of herbivory (two of five species), but the relationship was positive for *A. gerardii* and negative for *B. gracilis*. Relationships were variable in directionality among species, suggesting the influence of alternative drivers of herbivory were species‐specific (Figure 3). Herbivory on *B. dactyloides* showed the strongest patterns, increasing with GDD, precipitation, N, P, and soil pH (Figure 3), although the strongest predictor was GDD, as ranked by model *AICc* (Table S4). It may be argued that there is a multiple testing issue with our analyses. First, our main analysis and conclusions are based on an information‐theoretic approach to model comparison, and *P*‐values are reported for interested parties but were not relied on for our conclusions. Second, across the five‐grass species, we tested eight predictors with the expectation that 2 of these relationships would be significant by chance alone (at *p* = .05); instead, we detected seven significant relationships at the scale of species, suggesting that most relationships were not simply due to chance. Full statistical results are in Table S4.

## 4 DISCUSSION

### 4.1 Weak latitudinal patterns of leaf consumption

Leaf consumption increasd toward lower latitudes for dominant species of temperate grassland ecosystems, but this latitudinal pattern was weak. Several factors may account for interspecific variation in biogeographic patterns of herbivore damage. Species differed in both the mean and variance of herbivore damage (Figure 2), which can affect the ability to detect gradients. For example, species receiving low damage overall (e.g., *Bouteloua eriopoda*) had little variance in observed damage to be explained by latitude or any other geographic factors. This variance issue could be improved by increased sampling effort or by measuring complete consumption over the lifetime of individual plants, rather than standing damage at one plant phenological stage. However, such complete estimates are difficult to achieve in practice for long‐lived perennials, particularly over large geographic gradients (Anstett, Nunes, Baskett, & Kotanen, 2016). Sampling in multiple years would also produce more accurate estimates of herbivore damage. Potential caveats aside, our data suggest that a grass individual from low latitude will experience ~3% more leaf herbivore damage than an individual found more than 10º poleward in latitude (Figure 2a). Compared to studies where leaf area lost to herbivory over similar latitudinal gradients varied by ~12% (Kim, 2014) or ~35% (Baskett & Schemske, 2018), our results suggest insect herbivory over latitude is likely a minor driver of grass population dynamics. Additionally, grasslands have a unique evolutionary history with large mammalian herbivores that have all but disappeared in modern times (Frank, McNaughton, & Tracy, 1998), suggesting our results may have been different 150 years ago with natural grazing.

Our sampling design resulted in different geographic representation among grass species, which were each sampled at subsets of sites due to their natural distributions (Table S1). However, the variability among species in the correlates of herbivory was not likely created by differences in how much of each species’ range was sampled. We sampled roughly similar proportions of the North American latitudinal distributions for *B. dactyloides*, *B. gracilis*, *A. gerardi*, and *S. scoparium*, which have comparable ranges extending from southern Mexico into Canada. We sampled nearly the entire U.S. range of *B. eriopoda*, a species that showed no significant latitudinal pattern and has a range extending from northern Mexico to Wyoming (USDA and NRCS 2019).

For the two species with significant latitudinal clines in herbivory, higher damage at low latitude may be driven by unique patterns in the abundance or behavior of herbivores or by geographic variation in physiological plant traits that increase herbivore resistance (Cronin, Bhattarai, Allen, & Meyerson, 2015; Daehler & Strong, 1997; Dyer & Forister, 2019). It is well established that warm temperatures, along with long growing seasons can increase rates of herbivore consumption through direct physiological effects on ectothermic herbivores and by extending the length of time of herbivore exposure (e.g., Lemoine, Burkepile, & Parker, 2014). However, plant palatability may decrease at low latitudes to compensate for increases in leaf consumption (Hartley & DeGabriel, 2016; Pennings et al., 2007). While we lack data on palatability or leaf chemistry in our study, specific root length and specific leaf area (a trait that can positively correlate with palatability; Pérez‐Harguindeguy et al., 2003) may explain some of the herbivory variation in *B. gracilis*. Future studies could help to disentangle the influences of herbivore abundance, herbivore identity, and herbivore preferences (Dyer & Forister, 2019; Pennings et al., 2009).

### 4.2 Abiotic factors explained geographic variation in herbivory

Our findings highlight the issue that latitude is, biologically, an arbitrary variable that may encapsulate different abiotic or biotic drivers for different species (e.g., Hawkins & Diniz 2004, Lynn & Fridley, 2019). In our study, several site‐level climatic and edaphic factors predicted variation in herbivory, but in every case, these effects were plant species‐specific. Thus, our results demonstrate that the proximate factors that correlate with geographic variation in herbivore damage vary even among closely related plant taxa. In addition, we did not measure an important potential driver of herbivore damage, insect herbivore abundance, and so our predictions of herbivory could have been improved by incorporating unmeasured variables.

Contrary to several prior studies, our results suggest that precipitation, rather than temperature, was a better predictor of herbivory (Anstett et al., 2014; Zhang et al., 2016). Past work has shown that precipitation may affect plant–herbivore interactions via direct effects on insect herbivores or through indirect, plant‐mediated effects (reviewed in Barnett and Facey, 2016). For example, Moreira et al. (2018) observed an indirect effect of precipitation on leaf damage for an oak species. Similar to our study, Moreira et al. (2018) found a positive effect of precipitation on herbivory that was mediated by reduced plant chemical defenses. In our study, precipitation explained variation in herbivory for three of the five dominant C_4_ grasses (Table 3) and was the only climatic factor to influence herbivory on more than one species. The literature suggests that insect herbivory increases with precipitation (e.g., Moreira et al., 2015; Shure, Mooreside, & Ogle, 1998), which we generally found, with the exception of *S. scoparium*. For *S. scoparium*, higher herbivory at drier sites may have fitness consequences if aridity effects scale with drought effects, where heavy defoliation under drought can reduce the growth rate and biomass of grasses (Zhao, Chen, & Lin, 2008).

Herbivory also varied with additional climatic and edaphic factors, suggesting plant species‐specific controls on the biogeography of herbivory. Herbivory increased with GDD in *B. dactyloides*, which had the expected latitudinal cline in herbivory. This pattern was consistent with the hypothesis that longer growing seasons at low latitudes result in more herbivory than shorter seasons at high latitudes (Coley & Barone, 1996). Analyses of edaphic variables suggested that higher nutrient availability may also increase herbivore damage in some grasses (specifically *B. dactyloides*), perhaps by increasing plant nutrient content or altering belowground interactions. Abiotic conditions such as soil fertility have been shown to influence plant traits and herbivore nutrition in other systems (reviewed by Moles, Wallis, et al., 2011, see also Lynn & Fridley, 2019).

Although past work has uncovered climatic time lag effects in plant–pollinator interactions (e.g., Boggs & Inouye, 2012), tri‐trophic interactions (e.g., Post & Forchhammer, 2001), and vegetation dynamics (e.g., Weiss, Gutzler, Coonrod, & Dahm, 2004), less is known about climate windows, time lags, or the role of extreme climatic events in the geographical patterns of plant–herbivore interactions. We did not find strong evidence that climate windows varied in their ability to explain latitudinal variation in herbivory. However, for most grasses, the 30‐year average precipitation was the poorest predictor of herbivory compared to the three‐year average or precipitation during the year of sampling, suggesting that plants and herbivores respond more strongly to recent precipitation regimes. In contrast, all time windows for temperature (growing degree days) were equivalent in predicting herbivory, likely because year‐to‐year temperature variation is much smaller relative to year‐to‐year precipitation variation.

### 4.3 Plant traits can predict levels of herbivory, where patterns were plant species‐specific

Plant populations vary in traits that influence damage, such as defensive or nutritional traits (e.g., Hartley & DeGabriel, 2016), as well as leaf longevity, which can influence the cost of damage. In coastal salt marshes, for example, low‐latitude plants are better defended than high‐latitude plants, but nevertheless experience greater herbivory due to high densities of herbivores (e.g. Pennings et al., 2009). Variation in plant traits could not only amplify or counteract latitudinal gradients in damage, but also makes percentage damage an imperfect measure of herbivory intensity: The same amount of leaf area removed likely differs in its fitness cost among plant individuals and populations (Lim et al., 2015). For instance, plants with greater fine root SRL generally have greater nutrient/water uptake that could promote foliar nutrition (Ryser, 1998; Pérez‐Harguindeguy et al., 2013) and thereby increase herbivore damage. Alternatively, aboveground herbivory may alter SRL. For example, Thorne and Frank (2009) found that clipping leaves increased SRL in one of four plant species they examined. Although there was no significant latitudinal pattern in herbivory for *A. gerardii*, this species had higher herbivory with larger SRL. This observational evidence may help to link belowground investment strategies and aboveground trophic interactions. For *B. gracilis*, we found populations with higher SLA had more herbivore damage, and populations with high SRL had less herbivore damage. Some studies have reported a positive relationship, where thicker leaves (low SLA) are less palatable to insect herbivores (Pérez‐Harguindeguy et al., 2003). Additional trait‐based studies may help to resolve whether plant populations at low‐latitude sites are less palatable than those at higher latitudes or if geographic variation is driven more strongly by herbivore population densities. However, Kim (2014) found no relationship between plant resistance and herbivore damage for two species that displayed different latitudinal patterns in herbivory.

## 5 CONCLUSION

Here, we show that latitudinal gradients in herbivore damage generally occur in temperate grasslands but are weak among dominant, C_4_ perennial grass species. Several abiotic factors correlated with herbivory, although the magnitude and direction of their influence varied among grass species. Among these factors, 2015 precipitation and SRL were the strongest abiotic and trait correlates of herbivory. Our study suggests that higher temperatures and increased variation in precipitation with climate change in North American grassland ecosystems could have species‐specific effects on plant–herbivore interactions, thereby exacerbating or minimizing fitness effects of herbivory on grass populations.

## CONFLICT OF INTEREST

The authors have no conflicts of interest to declare.

## AUTHOR CONTRIBUTIONS

D.R.K.: Fieldwork; data analysis; manuscript writing‐review & editing. J.S.L.: Data analysis; manuscript writing‐review & editing. S.C.P.: Conceptualization and design of experiments; manuscript writing‐review & editing. L.A.S.: Fieldwork; manuscript writing‐review & editing. M.D.S.: Manuscript writing‐review & editing. J.A.R.: Conceptualization and design of experiments; fieldwork; data analysis; manuscript writing‐review & editing.

## Supporting information

Supplementary Material

## Data Availability

Data were deposited at the Environmental Data Initiative (https://environmentaldatainitiative.org) with https://doi.org/10.6073/pasta/910304380fc270f138f6b0f4307a5dcc.
