# Supplementary material for: Weak latitudinal gradients in insect herbivory for dominant rangeland grasses of North America
Source: Ecol Evol. 2020 May 26;10(13):6385–94. doi: 10.1002/ece3.6374 (PMC7381578; doi:10.1002/ece3.6374)
Supplement: Supplementary file 1 — Supplementary Material [file ECE3-10-6385-s001.docx]

**Appendices**

**Weak latitudinal gradients in insect herbivory for dominant rangeland grasses of North America**

Dylan R. Kent, Joshua S. Lynn, Steven C. Pennings, Lara A. Souza, Melinda D. Smith, and

Jennifer A. Rudgers

**Appendix Tables**

**Table S1** Study site information. Each site is represented by a site acronym. Survey type is either Latitudinal gradients (LAT) or EDGE plots (EDGE), which were the extra plots mentioned in the main text. Individual species were sampled where they naturally occurred, at local subsites within each site (designated A-C), each with Latitude, Longitude, and Elevation (m). In addition to the date of collection, we report the growing degree days (GDD) based on the 30-y climate average at each site. Number of individuals sampled for each species are shown with species acronyms: ANGE: *Andropogon gerardii*, BOER: *B. eriopoda*, BOGR: *B. gracilis*, BUDA: *B dactyloides*, and SCSC: *Schizachyrium scoparium*.

| **Gradient** | **Site** | **Survey** | **Sub**  **Site** | **Date Collected** | **GDD** | **Latitude** | **Longitude** | **Elevation** | **ANGE** | **BOER** | **BOGR** | **BUDA** | **SCSC** |
| --- | --- | --- | --- | --- | --- | --- | --- | --- | --- | --- | --- | --- | --- |
| East | SCP | LAT | A | 8/7/2015 | 2655 | 40.69482 | -96.85447 | 406 | 12 | 0 | 0 | 0 | 0 |
| East | SCP | LAT | B | 8/7/2015 | 2655 | 40.69234 | -96.85299 | 406 | 0 | 0 | 12 | 0 | 12 |
| East | ONF | LAT | A | 7/18/2015 | 2688 | 39.80954 | -94.13184 | 208 | 12 | 0 | 0 | 0 | 12 |
| East | KNZ | EDGE | A | 8/4/2015 | 2818 | 39.0852 | -96.5553 | 860 | 20 | 0 | 0 | 0 | 20 |
| East | KNZ | LAT | A | 8/3/2015 | 2792 | 39.0745 | -96.6036 | 860 | 12 | 0 | 12 | 12 | 12 |
| East | NWP | LAT | A | 7/17/2015 | 2599 | 36.0417 | -94.8137 | 250 | 12 | 0 | 0 | 0 | 12 |
| East | SFA | LAT | A | 7/4/2015 | 2648 | 31.09185 | -94.26535 | 74.7 | 0 | 0 | 0 | 0 | 12 |
| East | UHC | LAT | A | 6/29/2015 | 2652 | 29.39087 | -95.03443 | -19.8 | 12 | 0 | 0 | 0 | 12 |
| Middle | FCP | LAT | A | 7/20/2015 | 2429 | 36.0235 | -99.9439 | 635 | 0 | 0 | 12 | 0 | 12 |
| Middle | FCP | LAT | B | 7/20/2015 | 2429 | 36.0235 | -99.9439 | 635 | 0 | 0 | 0 | 12 | 0 |
| Middle | LAR | LAT | A | 8/9/2015 | 2451 | 40.66462 | -98.9063 | 632 | 12 | 0 | 12 | 0 | 12 |
| Middle | HAR | LAT | A | 8/5/2015 | 2710 | 39.0878 | -99.1559 | 613 | 12 | 0 | 12 | 12 | 12 |
| Middle | HAR | EDGE | A | 8/5/2015 | 2710 | 39.0878 | -99.1559 | 613 | 0 | 0 | 20 | 6 | 4 |
| Middle | KAE | LAT | B | 7/16/2015 | 2639 | 34.98053 | -97.52155 | 335 | 0 | 0 | 0 | 12 | 0 |
| Middle | KAE | LAT | A | 7/16/2015 | 2639 | 34.9779 | -97.5228 | 335 | 12 | 0 | 0 | 0 | 12 |
| Middle | CAD | LAT | B | 7/6/2015 | 2635 | 33.3087 | -97.60635 | 351.1 | 0 | 0 | 25 | 0 | 0 |
| Middle | CAD | LAT | A | 7/5/2015 | 2606 | 33.30862 | -97.605317 | 334.7 | 0 | 0 | 0 | 12 | 0 |
| Middle | CAD | LAT | C | 7/6/2015 | 2635 | 33.30787 | -97.6054167 | 339.2 | 12 | 0 | 0 | 0 | 12 |
| Middle | LBJ | LAT | A | 7/1/2015 | 2649 | 30.1847 | -97.8675 | 250.2 | 12 | 0 | 0 | 0 | 0 |
| Middle | LBJ | LAT | C | 7/2/2015 | 2676 | 30.18331 | -97.8768 | 231 | 0 | 0 | 0 | 12 | 0 |
| Middle | LBJ | LAT | B | 7/1/2015 | 2649 | 30.18225 | -97.874517 | 249.9 | 0 | 0 | 0 | 0 | 12 |
| West | HPG | EDGE | A | 8/25/2015 | 2206 | 41.122 | -104.5313 | 1930 | 0 | 0 | 20 | 0 | 0 |
| West | HPG | LAT | A | 8/24/2015 | 2206 | 41.122 | -104.5313 | 1930 | 0 | 0 | 12 | 12 | 0 |
| West | CPR | EDGE | A | 8/23/2015 | 2564 | 40.501 | -104.4545 | 1640 | 0 | 0 | 20 | 0 | 0 |
| West | CPR | LAT | A | 8/23/2015 | 2564 | 40.501 | -104.4545 | 1640 | 0 | 0 | 12 | 12 | 0 |
| West | RNF | LAT | A | 9/4/2015 | 1881 | 38.1882 | -106.514975 | 2747 | 0 | 0 | 12 | 0 | 0 |
| West | BLM | LAT | A | 9/4/2015 | 1881 | 37.62755 | -106.253289 | 2388 | 0 | 0 | 12 | 0 | 0 |
| West | BLM | LAT | B | 9/4/2015 | 1881 | 37.61693 | -106.262403 | 2374 | 0 | 0 | 0 | 0 | 12 |
| West | CNF | LAT | A | 9/5/2015 | 2633 | 36.23189 | -106.37604 | 1864 | 0 | 12 | 12 | 0 | 0 |
| West | CNF | LAT | B | 9/5/2015 | 2633 | 36.22032 | -106.37158 | 1834 | 0 | 0 | 0 | 0 | 12 |
| West | SEV | EDGE | A | 8/23/2015 | 3504 | 34.34214 | -106.62261 | 1645 | 0 | 20 | 20 | 0 | 0 |
| West | SEV | LAT | A | 8/23/2015 | 3504 | 34.34214 | -106.62261 | 1645 | 0 | 12 | 12 | 0 | 0 |
| West | SEV | EDGE | B | 8/23/2015 | 3504 | 34.33731 | -106.728392 | 1645 | 0 | 20 | 0 | 0 | 0 |
| West | GNF | LAT | A | 8/29/2015 | 2840 | 32.79587 | -108.18346 | 1881 | 0 | 12 | 12 | 0 | 0 |
| West | GNF | LAT | B | 8/30/2015 | 2860 | 32.74914 | -108.2876 | 1825 | 0 | 0 | 0 | 0 | 12 |
| West | FMT | LAT | B | 8/30/2015 | 3788 | 32.16806 | -107.75105 | 1311 | 0 | 0 | 12 | 0 | 0 |
| West | FMT | LAT | A | 8/29/2015 | 3764 | 32.03624 | -107.64451 | 1434 | 0 | 12 | 0 | 0 | 0 |
| West | GMT | LAT | A | 7/14/2015 | 2395 | 31.95908 | -104.760016 | 1551.4 | 0 | 12 | 12 | 0 | 0 |
| West | DMT | LAT | C | 7/17/2015 | 2528 | 30.69382 | -104.12415 | 1823.9 | 0 | 0 | 0 | 0 | 12 |
| West | DMT | LAT | B | 7/16/2015 | 2628 | 30.34758 | -104.039516 | 1466.4 | 0 | 0 | 12 | 0 | 0 |
| West | DMT | LAT | A | 7/16/2015 | 2628 | 30.34736 | -104.046683 | 1494.4 | 0 | 12 | 0 | 0 | 0 |
| West | BNP | LAT | B | 7/15/2015 | 2633 | 29.27425 | -103.286083 | 1705.4 | 0 | 0 | 12 | 0 | 0 |
| West | BNP | LAT | A | 7/15/2015 | 3039 | 29.23005 | -103.378316 | 1200.9 | 0 | 12 | 0 | 0 | 0 |
|  |  |  |  |  |  |  |  |  |  |  |  |  |  |

**Table S2** Results from models examining latitudinal gradients in mean-site herbivory across all focal species and for individual taxa. *P*-values are from Wald chi-square tests (*χ^2^*; see Methods), and values of *P* < 0.05 are shown in bold. *Marginal R^2^* only consider the dependent variables while *Conditional R^2^* incorporate the variance explained by random effects. *P*-intercept and *P*-slope are from Wald chi-square tests. For *B. eriopoda*, models were fit without random effects so the “conditional” *R^2^* is an *adjusted R^2^* and *P*-values are from F-tests. *DF* are residual degrees of freedom.

| *Species* | *Model term* | *P-value* | *χ^2^* | *Marginal R^2^* | *Conditional R^2^* | *df* |
| --- | --- | --- | --- | --- | --- | --- |
| All | intercept | 0.649 | 0.206 | 0.123 | 0.334 | 63 |
|  | latitude | **0.003** | 9.038 |  |  |  |
| *Andropogon* | intercept | 0.342 | 0.901 | 0.053 | 0.525 | 7 |
| *gerardii* | latitude | 0.295 | 1.097 |  |  |  |
| *Bouteloua* | intercept | 0.773 | 0.049 | 0.040 |  | 7 |
| *eriopoda* | latitude | 0.581 | 0.182 |  |  |  |
| *Bouteloua* | intercept | 0.647 | 0.210 | 0.253 | 0.252 | 17 |
| *gracilis* | latitude | **0.009** | 6.733 |  |  |  |
| *Bouteloua* | intercept | 0.080 | 3.059 | 0.575 | 0.591 | 5 |
| *dactyloides* | latitude | **0.001** | 10.697 |  |  |  |
| *Schizachyrium* | intercept | 0.086 | 2.954 | 0.000 | 0.180 | 14 |
| *scoparium* | latitude | 0.984 | 0.000 |  |  |  |

**Table S3** Results of models ranking the effects of climatic windows on herbivory. Predictors are listed in order from the best model to worst based on the *AICc* criterion. Models have statistically similar fits if Δ*AICc* <2. Weights can be interpreted as the probability that a given model is the most predictive out of the candidate set of models. *Marginal R^2^* only consider the dependent variables while *Conditional R^2^* incorporate the variance explained by random effects. *P*-intercept and *P*-slope are from Wald chi-square tests and values of *P* < 0.05 are shown in bold. For *B. eriopoda*, models were fit without random effects so the “conditional” *R^2^* is an *adjusted R^2^* and *P*-values are from F-tests. *DF* are residual degrees of freedom.

| *Species* | *Climate variable* | *Climate window* | *AICc* | *ΔAICc* | *Weights* | *Marginal R^2^* | *Conditional R^2^* | *P-intercept* | *P-slope* | *df* |
| --- | --- | --- | --- | --- | --- | --- | --- | --- | --- | --- |
| all | GDD | 3 | 200.2 | 0.0 | 0.41 | 0.04 | 0.36 | **<0.001** | 0.109 | 62 |
|  |  | 2015 | 200.5 | 0.3 | 0.35 | 0.04 | 0.37 | **<0.001** | 0.141 | 62 |
|  |  | 30 | 201.3 | 1.1 | 0.24 | 0.03 | 0.38 | **<0.001** | 0.215 | 62 |
|  | PPT | 30 | 199.1 | 0.0 | 0.41 | 0.01 | 0.38 | **<0.001** | 0.554 | 62 |
|  |  | 3 | 199.1 | 0.0 | 0.40 | 0.03 | 0.39 | **<0.001** | 0.339 | 62 |
|  |  | 2015 | 200.6 | 1.5 | 0.19 | 0.00 | 0.37 | **<0.001** | 0.876 | 62 |
| *Andropogon* | GDD | 3 | 33.6 | 0.0 | 0.36 | 0.09 | 0.37 | **<0.001** | 0.232 | 7 |
| *gerardii* |  | 2015 | 33.8 | 0.2 | 0.33 | 0.14 | 0.56 | **<0.001** | 0.267 | 7 |
|  |  | 30 | 34.0 | 0.4 | 0.30 | 0.06 | 0.38 | **<0.001** | 0.308 | 7 |
|  | PPT | 2015 | 32.5 | 0.0 | 0.54 | 0.14 | 0.56 | **<0.001** | 0.081 | 7 |
|  |  | 3 | 33.9 | 1.4 | 0.26 | 0.07 | 0.51 | **<0.001** | 0.261 | 7 |
|  |  | 30 | 34.5 | 2.0 | 0.20 | 0.06 | 0.53 | **<0.001** | 0.377 | 7 |
| *Bouteloua* | GDD | 2015 | 28.5 | 0.0 | 0.36 |  | -0.07 | 0.068 | 0.527 | 7 |
| *eriopoda* |  | 3 | 28.6 | 0.1 | 0.34 |  | -0.09 | 0.080 | 0.586 | 7 |
|  |  | 30 | 28.8 | 0.3 | 0.30 |  | -0.12 | 0.184 | 0.695 | 7 |
|  | PPT | 2015 | 28.9 | 0.0 | 0.34 |  | -0.13 | 0.052 | 0.776 | 7 |
|  |  | 3 | 29.0 | 0.1 | 0.33 |  | -0.13 | **0.015** | 0.830 | 7 |
|  |  | 30 | 29.0 | 0.1 | 0.32 |  | -0.14 | **0.038** | 0.969 | 7 |
| *Bouteloua* | GDD | 30 | 68.0 | 0.0 | 0.36 | 0.02 | 0.02 | **<0.001** | 0.575 | 17 |
| *gracilis* |  | 3 | 68.1 | 0.1 | 0.33 | 0.01 | 0.01 | **<0.001** | 0.683 | 17 |
|  |  | 2015 | 68.3 | 0.3 | 0.31 | 0.00 | 0.00 | **<0.001** | 0.848 | 17 |
|  | PPT | 3 | 64.2 | 0.0 | 0.55 | 0.18 | 0.18 | **<0.001** | **0.034** | 17 |
|  |  | 30 | 65.5 | 1.3 | 0.29 | 0.13 | 0.13 | **<0.001** | 0.086 | 17 |
|  |  | 2015 | 66.6 | 2.4 | 0.17 | 0.08 | 0.08 | **<0.001** | 0.189 | 17 |
| *Bouteloua* | GDD | 3 | 34.2 | 0.0 | 0.48 | 0.63 | 0.63 | **<0.001** | **<0.001** | 5 |
| *dactyloides* |  | 2015 | 34.8 | 0.6 | 0.34 | 0.60 | 0.60 | **<0.001** | **0.001** | 5 |
|  |  | 30 | 36.1 | 1.9 | 0.18 | 0.53 | 0.53 | **<0.001** | **0.003** | 5 |
|  | PPT | 2015 | 37.3 | 0.0 | 0.56 | 0.40 | 0.48 | **<0.001** | **0.018** | 5 |
|  |  | 3 | 38.4 | 1.1 | 0.33 | 0.27 | 0.46 | **<0.001** | 0.070 | 5 |
|  |  | 30 | 40.4 | 3.1 | 0.12 | 0.09 | 0.36 | **<0.001** | 0.397 | 5 |
| *Schizachyrium* | GDD | 30 | 52.4 | 0.0 | 0.41 | 0.08 | 0.08 | **0.032** | 0.213 | 14 |
| *scoparium* |  | 2015 | 53.0 | 0.6 | 0.31 | 0.05 | 0.06 | **0.050** | 0.346 | 14 |
|  |  | 3 | 53.1 | 0.7 | 0.29 | 0.04 | 0.06 | **0.031** | 0.413 | 14 |
|  | PPT | 3 | 47.9 | 0.0 | 0.61 | 0.29 | 0.29 | **<0.001** | **0.008** | 14 |
|  |  | 2015 | 49.7 | 1.8 | 0.25 | 0.22 | 0.22 | **<0.001** | **0.031** | 14 |
|  |  | 30 | 50.9 | 3.0 | 0.14 | 0.16 | 0.16 | **<0.001** | 0.072 | 14 |

**Table S4** Results of models ranking the effects of climatic, edaphic, and trait predictors on herbivory. Predictors are listed in order from the best model to worst based on the *AICc* criterion. Models have statistically similar fits if Δ*AICc* <2. Weights can be interpreted as the probability that a given model is the most predictive out of the candidate set of models. *Marginal R^2^* only consider the dependent variables while *Conditional R^2^* incorporate the variance explained by random effects. *P*-intercept and *P*-slope are from Wald chi-square tests and values of *P* < 0.05 are shown in bold. For *B. eriopoda*, models were fit without random effects so the “conditional” *R^2^* is an *adjusted R^2^* and *P*-values are from F-tests. *DF* are residual degrees of freedom.

| *Species* | *Variable* | *AICc* | *ΔAICc* | *Weights* | *Marginal R^2^* | *Conditional R^2^* | *P-intercept* | *P-slope* | *df* |
| --- | --- | --- | --- | --- | --- | --- | --- | --- | --- |
| all | SRL | 180.5 | 0.0 | 0.41 | 0.08 | 0.35 | **<0.001** | **0.017** | 62 |
|  | pH | 181.9 | 1.4 | 0.20 | 0.06 | 0.36 | **<0.001** | **0.046** | 62 |
|  | gdd | 183.1 | 2.6 | 0.11 | 0.05 | 0.27 | **<0.001** | 0.105 | 62 |
|  | P | 183.8 | 3.3 | 0.08 | 0.03 | 0.31 | **<0.001** | 0.168 | 62 |
|  | N | 184.4 | 3.9 | 0.06 | 0.02 | 0.31 | **<0.001** | 0.251 | 62 |
|  | SOM | 184.4 | 4.0 | 0.06 | 0.02 | 0.32 | **<0.001** | 0.303 | 62 |
|  | SLA | 184.5 | 4.1 | 0.05 | 0.01 | 0.30 | **<0.001** | 0.344 | 62 |
|  | ppt | 185.4 | 4.9 | 0.04 | 0.00 | 0.29 | **<0.001** | 0.865 | 62 |
| *Andropogon* | SRL | 29.9 | 0.0 | 0.48 | 0.34 | 0.52 | **<0.001** | **0.011** | 7 |
| *gerardii* | ppt | 32.5 | 2.6 | 0.13 | 0.14 | 0.56 | **<0.001** | 0.081 | 7 |
|  | pH | 33.0 | 3.2 | 0.10 | 0.12 | 0.59 | **<0.001** | 0.113 | 7 |
|  | SOM | 33.4 | 3.6 | 0.08 | 0.13 | 0.32 | **<0.001** | 0.190 | 7 |
|  | gdd | 33.6 | 3.7 | 0.07 | 0.09 | 0.37 | **<0.001** | 0.232 | 7 |
|  | P | 34.3 | 4.5 | 0.05 | 0.04 | 0.44 | **<0.001** | 0.404 | 7 |
|  | SLA | 34.5 | 4.6 | 0.05 | 0.03 | 0.37 | **<0.001** | 0.474 | 7 |
|  | N | 34.5 | 4.7 | 0.05 | 0.03 | 0.40 | **<0.001** | 0.498 | 7 |
| *Bouteloua* | SLA | 27.2 | 0.0 | 0.23 |  | 0.07 | **<0.001** | 0.241 | 7 |
| *eriopoda* | pH | 27.9 | 0.8 | 0.16 |  | -0.01 | **<0.001** | 0.368 | 7 |
|  | N | 28.6 | 1.4 | 0.11 |  | -0.09 | **<0.001** | 0.567 | 7 |
|  | gdd | 28.6 | 1.5 | 0.11 |  | -0.09 | **<0.001** | 0.586 | 7 |
|  | SOM | 28.7 | 1.5 | 0.11 |  | -0.09 | **<0.001** | 0.595 | 7 |
|  | P | 28.9 | 1.7 | 0.10 |  | -0.12 | **<0.001** | 0.743 | 7 |
|  | ppt | 28.9 | 1.8 | 0.09 |  | -0.13 | **<0.001** | 0.776 | 7 |
|  | SRL | 29.0 | 1.8 | 0.09 |  | -0.13 | **<0.001** | 0.806 | 7 |
| *Bouteloua* | SRL | 63.1 | 0.0 | 0.46 | 0.23 | 0.23 | **<0.001** | **0.016** | 17 |
| *gracilis* | SLA | 65.6 | 2.4 | 0.14 | 0.13 | 0.13 | **<0.001** | 0.087 | 17 |
|  | pH | 65.6 | 2.4 | 0.14 | 0.13 | 0.13 | **<0.001** | 0.088 | 17 |
|  | ppt | 66.6 | 3.5 | 0.08 | 0.08 | 0.08 | **<0.001** | 0.189 | 17 |
|  | SOM | 67.0 | 3.8 | 0.07 | 0.06 | 0.06 | **<0.001** | 0.246 | 17 |
|  | P | 68.1 | 4.9 | 0.04 | 0.01 | 0.01 | **<0.001** | 0.642 | 17 |
|  | gdd | 68.1 | 5.0 | 0.04 | 0.01 | 0.01 | **<0.001** | 0.683 | 17 |
|  | N | 68.3 | 5.1 | 0.04 | 0.00 | 0.00 | **<0.001** | 0.872 | 17 |
| *Bouteloua* | gdd | 34.2 | 0.0 | 0.55 | 0.63 | 0.63 | **<0.001** | **<0.001** | 5 |
| *dactyloides* | P | 37.0 | 2.9 | 0.13 | 0.48 | 0.48 | **<0.001** | **0.007** | 5 |
|  | ppt | 37.3 | 3.2 | 0.11 | 0.40 | 0.48 | **<0.001** | **0.018** | 5 |
|  | N | 37.8 | 3.6 | 0.09 | 0.43 | 0.43 | **<0.001** | **0.014** | 5 |
|  | pH | 39.4 | 5.2 | 0.04 | 0.31 | 0.31 | **<0.001** | 0.056 | 5 |
|  | SRL | 40.1 | 5.9 | 0.03 | 0.07 | 0.60 | **<0.001** | 0.267 | 5 |
|  | SOM | 40.6 | 6.4 | 0.02 | 0.05 | 0.44 | **<0.001** | 0.495 | 5 |
|  | SLA | 40.9 | 6.8 | 0.02 | 0.01 | 0.40 | **<0.001** | 0.749 | 5 |
| *Schizachyrium* | ppt | 49.7 | 0.0 | 0.45 | 0.22 | 0.22 | **<0.001** | **0.031** | 14 |
| *scoparium* | pH | 52.2 | 2.5 | 0.13 | 0.09 | 0.09 | **<0.001** | 0.182 | 14 |
|  | gdd | 53.1 | 3.4 | 0.08 | 0.04 | 0.06 | **<0.001** | 0.413 | 14 |
|  | SLA | 53.4 | 3.7 | 0.07 | 0.01 | 0.11 | **<0.001** | 0.638 | 14 |
|  | SRL | 53.5 | 3.8 | 0.07 | 0.01 | 0.05 | **<0.001** | 0.682 | 14 |
|  | P | 53.5 | 3.8 | 0.07 | 0.01 | 0.07 | **<0.001** | 0.746 | 14 |
|  | N | 53.6 | 3.9 | 0.06 | 0.00 | 0.08 | **<0.001** | 0.971 | 14 |
|  | SOM | 53.6 | 3.9 | 0.06 | 0.00 | 0.09 | **<0.001** | 0.977 | 14 |

**Table S5** Random effect variance terms for each model grouped by species and predictor variable with columns representing estimates for a given random effect. Note, variance terms for PPT and GDD are from the 2015 and three-year climate windows as in the model selection analysis.

| *Species* | *Variable* | *Gradient* | *Site* | *Species* |
| --- | --- | --- | --- | --- |
| all | Latitude | 0.04837 | 0.06144 | 0.06618 |
|  | GDD | 0.08922 | 0.12180 | 0.06914 |
|  | PPT | 0.08563 | 0.15876 | 0.08260 |
|  | N | 0.04646 | 0.13559 | 0.05310 |
|  | P | 0.03907 | 0.13695 | 0.04568 |
|  | pH | 0.00000 | 0.15714 | 0.07951 |
|  | SOM | 0.0000001992 | 0.17400 | 0.06268 |
|  | SLA | 0.008608 | 0.180884 | 0.038626 |
|  | SRL | 0.00000006603 | 0.01994 | 0.01545 |
| *Andropogon* | Latitude | 0.2919 |  |  |
| *gerardii* | GDD | 0.1140 |  |  |
|  | PPT | 0.2031 |  |  |
|  | N | 0.1685 |  |  |
|  | P | 0.1840 |  |  |
|  | pH | 0.2434 |  |  |
|  | SOM | 0.07822 |  |  |
|  | SLA | 0.1491 |  |  |
|  | SRL | 0.07121 |  |  |
| *Bouteloua* | Latitude | 0.0000 |  |  |
| *gracilis* | GDD | 0.0000 |  |  |
|  | PPT | 0.0000 |  |  |
|  | N | 0.0000 |  |  |
|  | P | 0.0000 |  |  |
|  | pH | 0.0000 |  |  |
|  | SOM | 0.0000 |  |  |
|  | SLA | 0.0000 |  |  |
|  | SRL | 0.0000 |  |  |
| *Bouteloua* | Latitude | 0.01679 |  |  |
| *dactyloides* | GDD | 0.000 |  |  |
|  | PPT | 0.06622 |  |  |
|  | N | 0.0000 |  |  |
|  | P | 0.0000 |  |  |
|  | pH | 0.0000 |  |  |
|  | SOM | 0.3602 |  |  |
|  | SLA | 0.3470 |  |  |
|  | SRL | 0.5543 |  |  |
| *Schizachyrium* | Latitude | 0.1347 |  |  |
| *scoparium* | GDD | 0.01546 |  |  |
|  | PPT | 0.0000 |  |  |
|  | N | 0.0000 |  |  |
|  | P | 0.04143 |  |  |
|  | pH | 0.04143 |  |  |
|  | SOM | 0.05717 |  |  |
|  | SLA | 0.05989 |  |  |
|  | SRL | 0.02467 |  |  |

**Table S6** Random slope terms in all species models do not improve fit of the model. Columns show AICc scores for each model for “intercept” random effects models and “slope” random effect for species in the models.

| *Variable* | *Intercept* | *Slope* |
| --- | --- | --- |
| Latitude | 184.1535 | 186.4479 |
| GDD | 183.0926 | 187.6374 |
| PPT | 185.3615 | 189.7026 |
| N | 184.3504 | 189.4139 |
| P | 183.7597 | 188.7955 |
| pH | 181.8797 | 185.9750 |
| SOM | 184.4436 | 189.4642 |
| SLA | 184.5395 | 189.8366 |
| SRL | 180.4569 | 183.2065 |
